# Indibulin dampens microtubule dynamics and produces synergistic antiproliferative effect with vinblastine in MCF-7 cells: Implications in cancer chemotherapy

**DOI:** 10.1038/s41598-018-30376-y

**Published:** 2018-08-17

**Authors:** Sonia Kapoor, Shalini Srivastava, Dulal Panda

**Affiliations:** 1 Department of Biosciences and Bioengineering, Indian Institute of Technology Bombay, Mumbai, 400076 India; 2 Amity Institute of Molecular Medicine and Stem Cell Research, Amity University, Noida, 201313 India

## Abstract

Indibulin, a synthetic inhibitor of tubulin assembly, has shown promising anticancer activity with a minimal neurotoxicity in preclinical animal studies and in Phase I clinical trials for cancer chemotherapy. Using time-lapse confocal microscopy, we show that indibulin dampens the dynamic instability of individual microtubules in live breast cancer cells. Indibulin treatment also perturbed the localization of end-binding proteins at the growing microtubule ends in MCF-7 cells. Indibulin reduced inter-kinetochoric tension, produced aberrant spindles, activated mitotic checkpoint proteins Mad2 and BubR1, and induced mitotic arrest in MCF-7 cells. Indibulin-treated MCF-7 cells underwent apoptosis-mediated cell death. Further, the combination of indibulin with an anticancer drug vinblastine was found to exert synergistic cytotoxic effects on MCF-7 cells. Interestingly, indibulin displayed a stronger effect on the undifferentiated neuroblastoma (SH-SY5Y) cells than the differentiated neuronal cells. Unlike indibulin, vinblastine and colchicine produced similar depolymerizing effects on microtubules in both differentiated and undifferentiated SH-SY5Y cells. The data indicated a possibility that indibulin may reduce chemotherapy-induced peripheral neuropathy in cancer patients.

## Introduction

**Figure 1:**
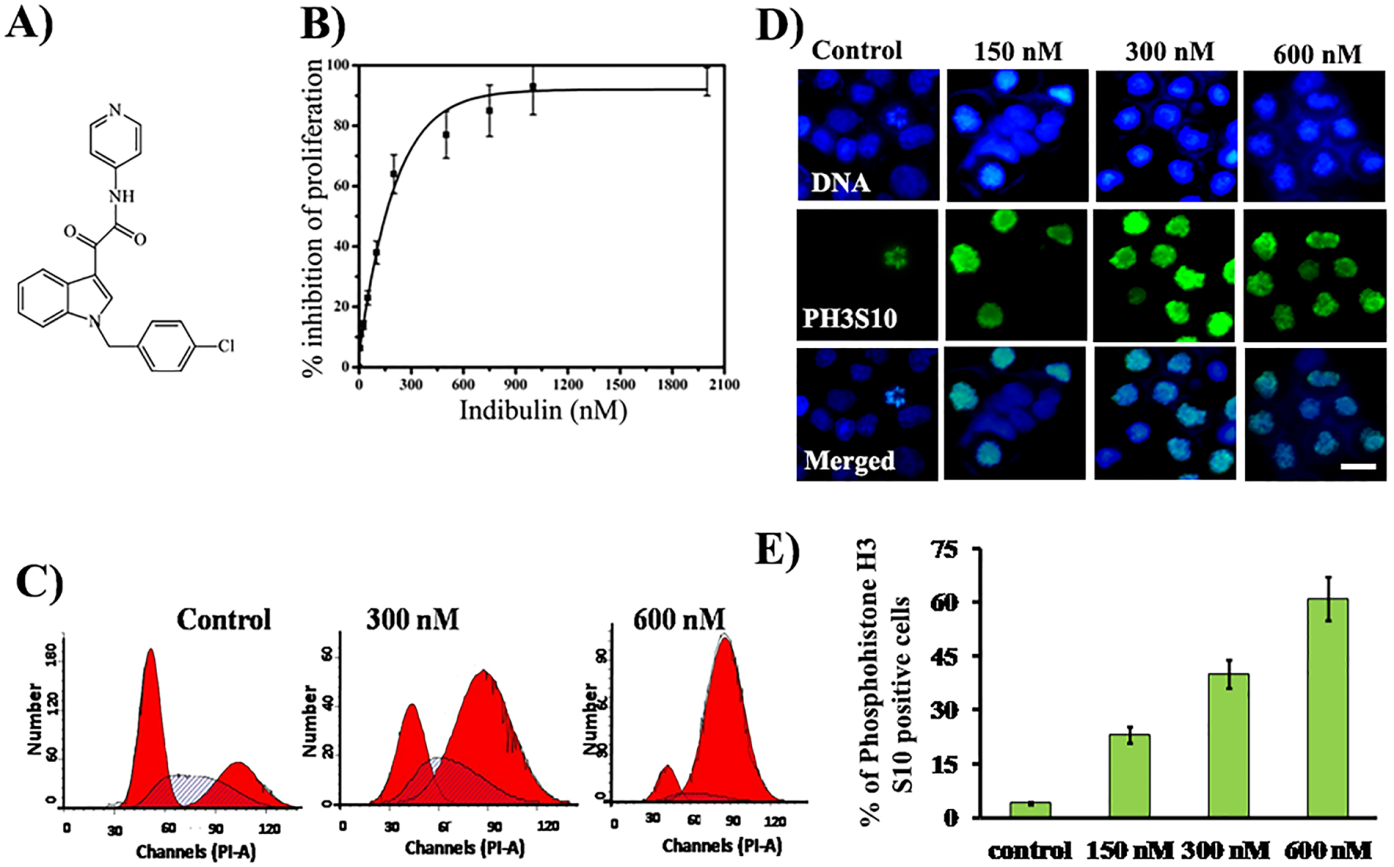
Indibulin inhibited the proliferation of MCF-7 cells and blocked the cell cycle progression at mitosis: (**A**) Structure of indibulin. (**B**) MCF-7 cells were treated with a vehicle or increasing concentrations of indibulin for 48 h. The inhibition of cell proliferation was determined by the SRB assay. Data are average of three independent experiments. (**C**) Cells were treated with vehicle or different concentrations (300 and 600 nM) of indibulin for 48 h and stained with propidium iodide. The DNA content of the cells was quantified by a flow cytometer and the data were analyzed using the Modfit LT program (Verity Software, ME, USA). The dark black lines show the fitting of the data by Modfit LT program and in each panel, the peaks correspond to G1 phase (left-side red peak), S phase (middle hashed line peak) and G2/M phase (right-side red peak). (**D**) Cells were treated with a vehicle or different concentrations of indibulin for 48 h and then stained with an antibody against phosphohistone H3 (S10) (green), a mitotic marker. DNA stained with Hoechst is shown in blue. Scale bar = 10 µm. (**E**) The histogram shows the percentage of phosphohistone H3 (S10) positive cells in the presence of a vehicle or different concentrations of indibulin. Three hundred cells were counted using Hoechst staining. Data are average of three independent experiments and error bar represents S.D.

Indibulin, *N*-(pyridin-4-yl)-[1-(4-chlorbenzyl)-indol-3-yl]-glyoxyl-amide (D-24851) (Fig. 1A) has been found to have a potent activity towards a wide variety of cancer cell lines including colon, ovarian, cervix, brain, and pancreatic cancer cell lines^1,2^. It also retained its efficacy in multidrug-resistant cells lines that were resistant towards paclitaxel, vincristine, or doxorubicin suggesting that indibulin might not be a substrate of P-gp pumps^1^. Indibulin has a peculiar advantage of oral applicability. It was found that an oral administration of indibulin for 2 weeks in rats (implanted with Yoshida AH13 sarcoma cells to form a tumor) induced a complete tumor remission^1^. Moreover, the curative doses of indibulin exerted no hematological toxicities and were well accepted with no apparent toxicity as indicated by maintenance of body weight of rats^1^. In human patients, the concentration of orally administered indibulin peaks in plasma after ~4 h of administration and remains above the lower limit of quantitation for about 96 h^3^, indicating its good bioavailability. The orally administered indibulin has been found to be well tolerated in human patients^4,5^, though further improvement in the formulation was needed. The most interesting and unique advantage of indibulin was shown to be its comparatively low neuronal toxicity. By using a coordination test (Rota-rod testing) on rats and by measuring peripheral neuronal conduction velocity (NCV) (both tests are indicators of the extent of neurotoxicity), it was shown that while vincristine and paclitaxel severely affected the behavioral performance of rats at their respective antitumoral efficacious doses, indibulin showed minimal effects indicating low neurotoxicity^1^. During the phase I clinical trials also, though a limited haematologic toxicity (≤grade 2) was observed, no major clinical symptoms of neurotoxicity were reported^4,5^. Further, indibulin exhibited promising activity against advanced and refractory solid tumors^4,5^. As a result, clinical trials have been carried out for evaluating its efficacy in the treatment of advanced solid tumors and metastatic breast cancer either alone or in combination with other chemotherapeutic agents including erlotinib and capecitabine (ClinicalTrials.gov Identifiers: NCT00591292; NCT00591136; NCT00591890; NCT00591383; NCT00591383; NCT00726687; NCT01113970). Several derivatives of D-24851 have been reported to display potent antiproliferative properties against different tumor cell types including non-small human lung cancer cells, leukemia P388 cells, human gastric, breast, and uterus cancer cells, and head and neck tumor^6–9^.

The antitumor activity of indibulin is believed to be primarily related to its effects on microtubules. Indibulin has been shown to depolymerize microtubules and to inhibit cell cycle progression at G2/M phase^1^. It has been suggested to bind to tubulin at a site other than the site of known microtubule depolymerizing agents^1^. However, the mode of interaction of indibulin to tubulin, and the mechanism of its anti-proliferative activity are far from clear.

In this study, we found that indibulin suppressed the dynamics of individual microtubules in live MCF-7 cells and inhibited the mitotic progression in these cells. Indibulin treatment activated spindle checkpoint proteins and induced apoptosis-mediated cell death. We present data suggesting that indibulin exerts a synergistic cytotoxic effect on breast cancer cells along with vinblastine. Our data also showed that indibulin exerted a differential effect on differentiated and undifferentiated neuronal cell lines and hence could lead to reduced neurotoxicity as compared to other anti-tubulin agents.

## Results

### Indibulin inhibited the proliferation of MCF-7 cells and blocked the cell cycle progression at mitosis

Indibulin inhibited the proliferation of MCF-7 cells with a half-maximal inhibitory concentration of 150 ± 13 nM (Fig. 1B). A flow cytometric analysis using propidium iodide staining suggested that indibulin treatment blocked the cells in the G_2_/M phase of the cell cycle (Fig. 1C). Furthermore, the percentage of phosphohistone H3 (S10) (a mitotic marker) positive cells increased from 4.1 ± 0.6% in control to 61 ± 5.3% in 600 nM indibulin treated cells indicating that indibulin blocked the progression of the cell cycle at mitosis (Fig. 1D,E).

### Indibulin depolymerized microtubules in MCF-7 cells

**Figure 2:**
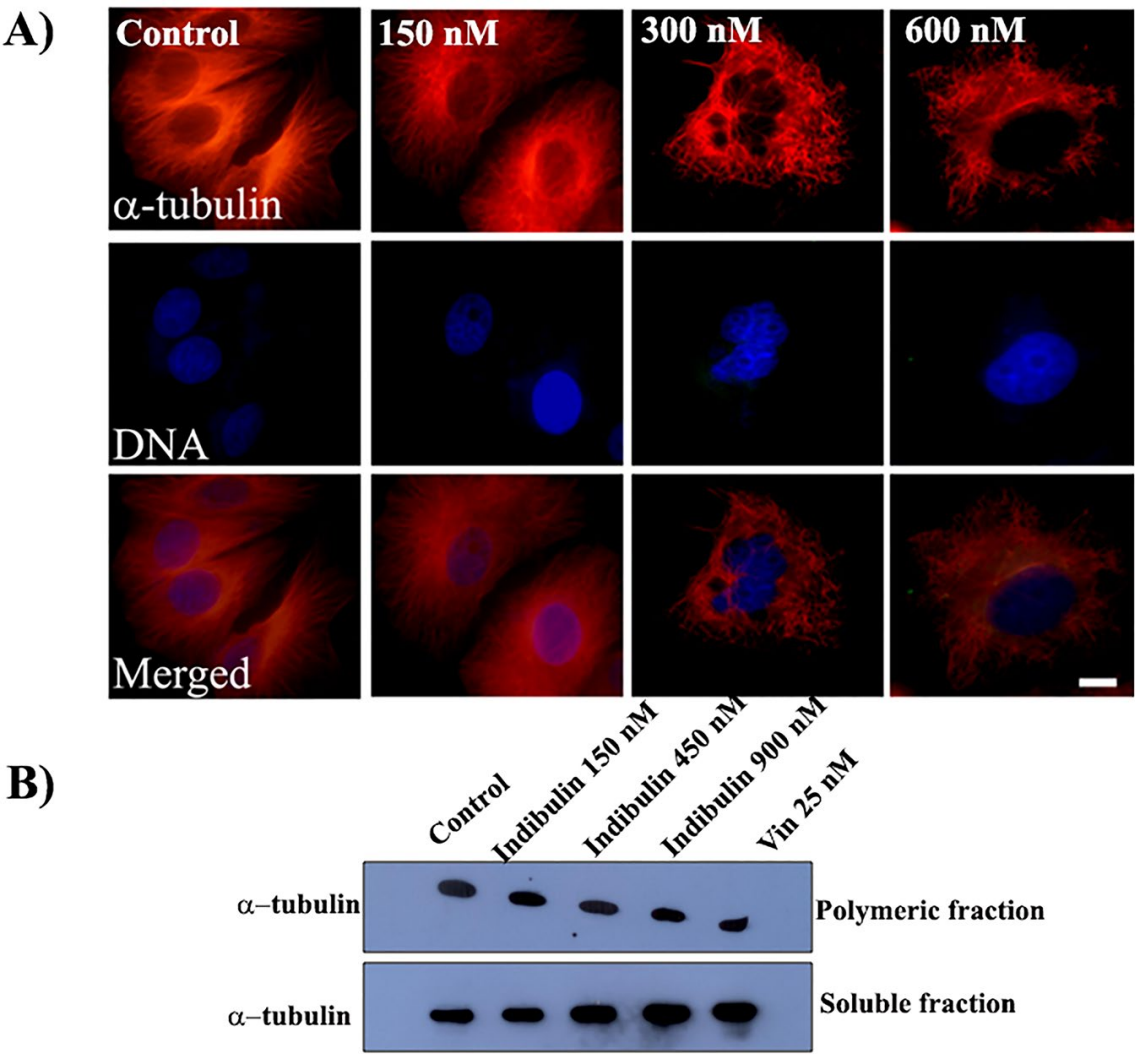
Effects of indibulin on interphase microtubules in MCF-7 cells: (**A**) Cells were incubated with vehicle or different concentrations of indibulin for 24 h and were then stained with an antibody against α-tubulin (red). DNA stained with Hoechst is shown in blue. Scale bar = 10 µm. (**B**) MCF-7 cells were treated with vehicle or 150, 450 and 900 nM indibulin (lanes 1–4, respectively) for 48 h. 25 nM vinblastine (lane 5) was used under similar experimental conditions as a control. Polymeric and soluble tubulin fractions were isolated, loaded separately on two different SDS-PAGEs and immunoblotted with the α-tubulin antibody.

We then determined the effect of indibulin on cellular microtubules. Indibulin treatment depolymerized microtubules in MCF-7 cells (Fig. 2A). Indibulin (150 nM) did not visibly perturb microtubule network in the interphase cells. However, the disruption of the microtubule network was clearly visible in the presence of 300 nM indibulin and most of the microtubules were depolymerized in the presence of 600 nM of the compound (Fig. 2A). The polymeric tubulin to soluble tubulin ratio was determined to be 2.3 ± 0.6, 1.6 ± 0.4 (p < 0.05), 1.1 ± 0.2 (p < 0.05), 0.9 ± 0.2 (p < 0.05) in the absence and presence of 150, 450 and 900 nM indibulin, respectively by Western blotting suggesting that indibulin treatment depolymerized microtubules in MCF-7 cells (Fig. 2B). Under similar conditions, as compared to control a ~3-fold decrease in the polymer to soluble tubulin ratio was observed in the presence of 25 nM vinblastine, a known microtubule depolymerizing agent.

**Figure 3:**
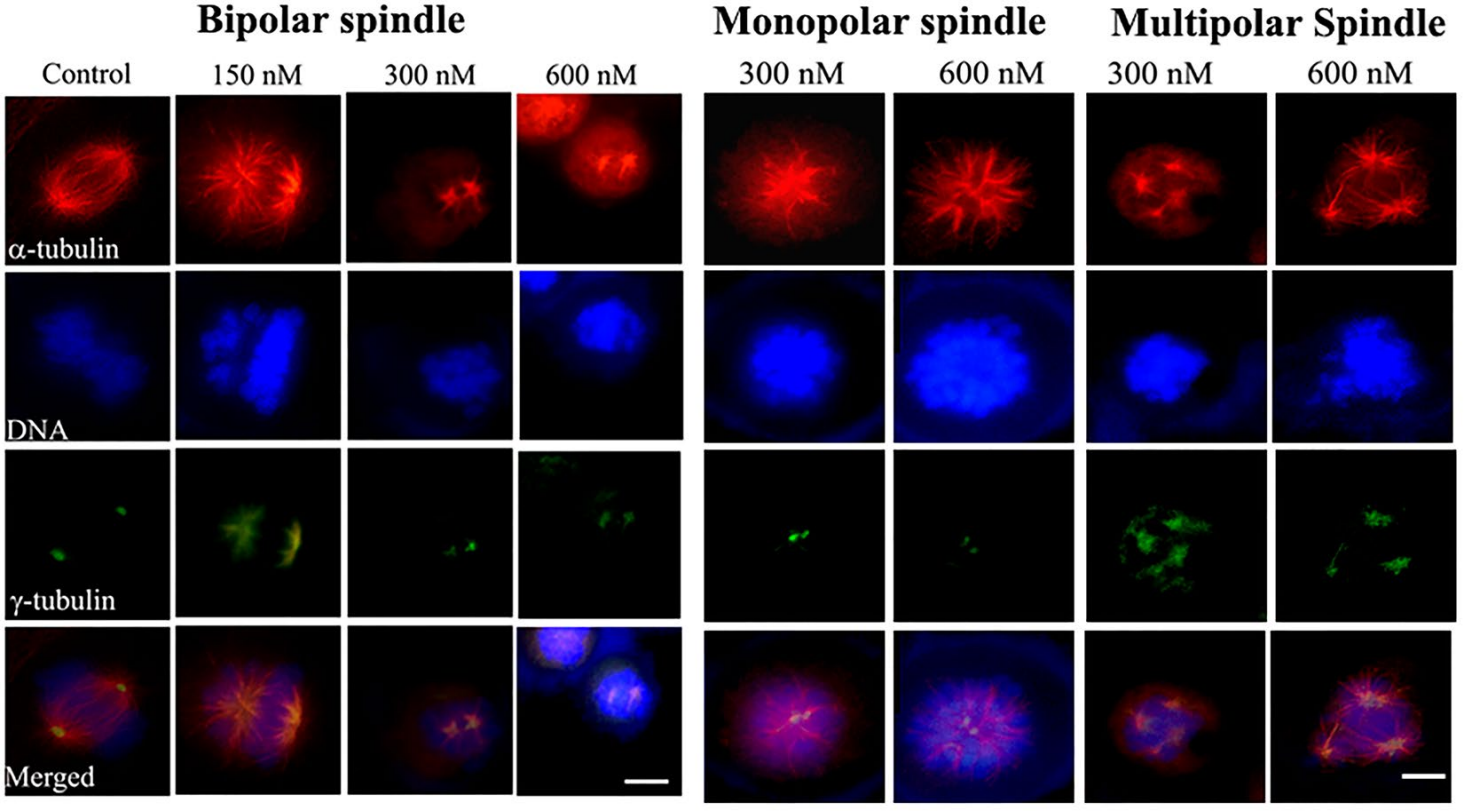
Effects of indibulin on mitotic MCF-7 cells: Cells were treated without and with different concentrations of indibulin and were stained with antibodies against α-tubulin (red) and γ-tubulin (green). Scale bar = 10 µm.

In the mitotic cells, chromosome congression defects were visible in several of the 150 nM indibulin treated cells and the mitotic microtubules were strongly disrupted in the presence of 300 and 600 nM indibulin (Fig. 3). Several monopolar and multipolar spindles were observed in cells treated with indibulin (Fig. 3). For example, 3, 26, 44 and 60% of the mitotic cells were found to be multi-polar and 3, 14, 33 and 36% of the mitotic cells were found to be monopolar in the absence or presence of 150, 300 and 600 nM indibulin, respectively. In addition, indibulin treatment significantly reduced the average spindle length (pole-to-pole distance determined by γ-tubulin staining) in the mitotic cells having a discernible bipolar spindle. For instance, the inter-centrosomal distance reduced from 11.5 ± 1.6 μm in control cells to 7.9 ± 0.8 (p ≤ 0.005), 4.7 ± 0.7 (p ≤ 0.005) and 3.9 ± 0.9 μm (p ≤ 0.005) in the presence of 150, 300 and 600 nM indibulin, respectively (n = 20 in each case) (Fig. 3). Further, the alignment of chromosomes on the metaphase plate was severely affected in 300 and 600 nM indibulin treated cells suggesting that indibulin treatment significantly perturbed proper spindle formation in MCF-7 cells (Fig. 3).

### Indibulin suppressed the dynamic instability of individual microtubules in live MCF-7 cells

**Figure 4:**
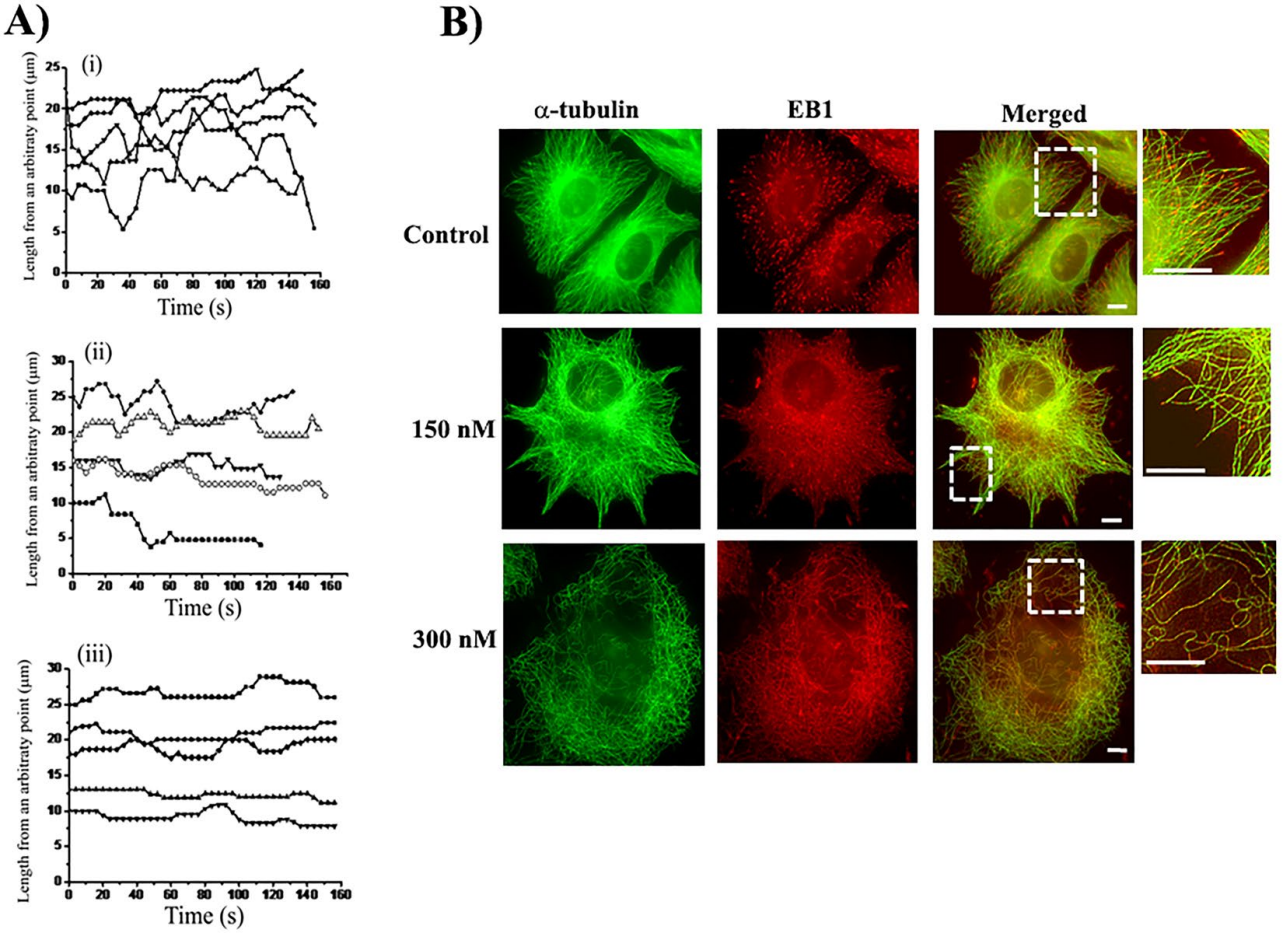
Indibulin suppressed microtubule dynamics in live MCF-7 cells: (**A**) Life history traces of microtubules of cells treated with (i) vehicle, (ii) 75 and (iii) 150 nM indibulin for 3 h. (**B**) Cells were treated with a vehicle or different concentrations (150 and 300 nM) of indibulin for 24 h and were processed for immunostaining with antibodies against EB1 (red) and α-tubulin (green). Boxed regions of merged panels are shown at higher magnification. Scale bar = 10 µm.

To examine the effect of indibulin on kinetic properties of microtubules, we transfected MCF-7 cells with EGFP-tubulin and followed the movements of microtubule plus ends using time-lapse fluorescence microscopy^10–12^. The life history traces of microtubules were constructed by plotting the lengths of the microtubules against time. Consistent with the previous reports^11–14^, microtubules in the vehicle-treated MCF-7 cells were found to be highly dynamic (Fig. 4A,i). In contrast, the growing and shortening excursions of plus ends of microtubules were severely curtailed in the presence of indibulin (Fig. 4A, ii and iii). Indibulin treatment strongly affected different parameters of dynamic instability of microtubules in MCF-7 cells (Table 1). In the presence of 150 nM (IC_50_) indibulin, the rates of growth and shortening states of microtubules were reduced by 37 and 53%, respectively. The % of time spent by microtubules in pause state increased from 38.3 ± 6.5% in control to 56.9 ± 7.4 (p ≤ 0.0005) and 71 ± 8.7% (p ≤ 0.0005) in 75 and 150 nM indibulin treated cells, respectively (Table 1). The time spent by microtubules in the growth and shortening phases strongly reduced in the presence of indibulin. Indibulin (75 nM) increased the length based catastrophe (a transition from a growing or a pause state to a shortening state)^12,15^ and rescue (a transition from a shortening to a growing or a pause state)^12,15^ frequencies by 105% (from 0.39 ± 0.14 to 0.8 ± 0.3 events/μm) and 63% (from 0.43 ± 0.2 to 0.7 ± 0.24 events/μm), respectively. The dynamicity (total change in microtubule length per unit time) was diminished by 35 and 71% in the presence of 75 and 150 nM indibulin, respectively suggesting that indibulin strongly suppressed the dynamic instability of microtubules (Table 1).

**Table 1:** Indibulin affected the parameters of dynamic instability of interphase microtubules in MCF-7 cells.

Parameters	Control	75 nM Indibulin	150 nM Indibulin
Growth rate (µm/min)	16.3 ± 2.4	13.2 ± 1.9^b^	10.2 ± 1.4^a^
Growth length (µm)	2.0 ± 0.6	1.4 ± 0.4^b^	0.83 ± 0.26^a^
Shortening rate (µm/min)	21.5 ± 3.8	15.1 ± 4.7^a^	10.1 ± 2.7^a^
Shortening length (µm)	2.9 ± 1.0	2.2 ± 0.6^b^	1.0 ± 0.3^a^
% time spent in growing	33.9 ± 6.2	21.1 ± 5.3^b^	15.0 ± 3.6^a^
% time spent in shortening	27.8 ± 4.0	22.0 ± 4.7^b^	14.0 ± 3.0^a^
% time spent in pause	38.3 ± 6.5	56.9 ± 7.4^a^	71.0 ± 8.7^a^
Dynamicity (µm/min)	10.4 ± 3.2	6.8 ± 2.6^b^	3.0 ± 1.2^a^
Catastrophe frequency (events/min)	2.8 ± 1.0	2.2 ± 0.4^c^	1.9 ± 0.6^a^
Rescue frequency (events/min)	8.3 ± 1.6	9.0 ± 1.0^d^	10.0 ± 2.0^d^
Catastrophe frequency (events/µm)	0.39 ± 0.14	0.8 ± 0.3^b^	1.5 ± 0.33^a^
Rescue frequency (events/µm)	0.43 ± 0.2	0.7 ± 0.24^b^	0.91 ± 0.27^a^

Data represent mean ± SD, n = 20 microtubules in each case. ^a^p ≤ 0.0005; ^b^p ≤ 0.005; ^c^p ≤ 0.05; ^d^Not significant.

### Indibulin perturbed the localization of EB1, a plus end-binding protein, at microtubule ends

EB1 is an important microtubule plus end-binding protein that localizes at the tip of the growing microtubules^16,17^. Since indibulin affected microtubule dynamics, we looked at its effect on the localization of endogenous EB1 by immunostaining. In vehicle-treated cells, EB1 was found to localize as comet at the ends of the microtubules growing towards the cell periphery (Fig. 4B). In cells treated with 150 nM indibulin, which suppressed microtubule dynamics but did not visibly affect microtubule network, the localization of the EB1 was perturbed and EB1 seemed to localize diffusely as dots at the ends of microtubules as well as in the cytoplasm. In the presence of 300 nM indibulin, EB1 was found to be mislocalized and appeared to be stained all along the length of the remaining microtubules (Fig. 4B).

### Indibulin activated mitotic checkpoints and induced apoptosis in MCF-7 cells

**Figure 5:**
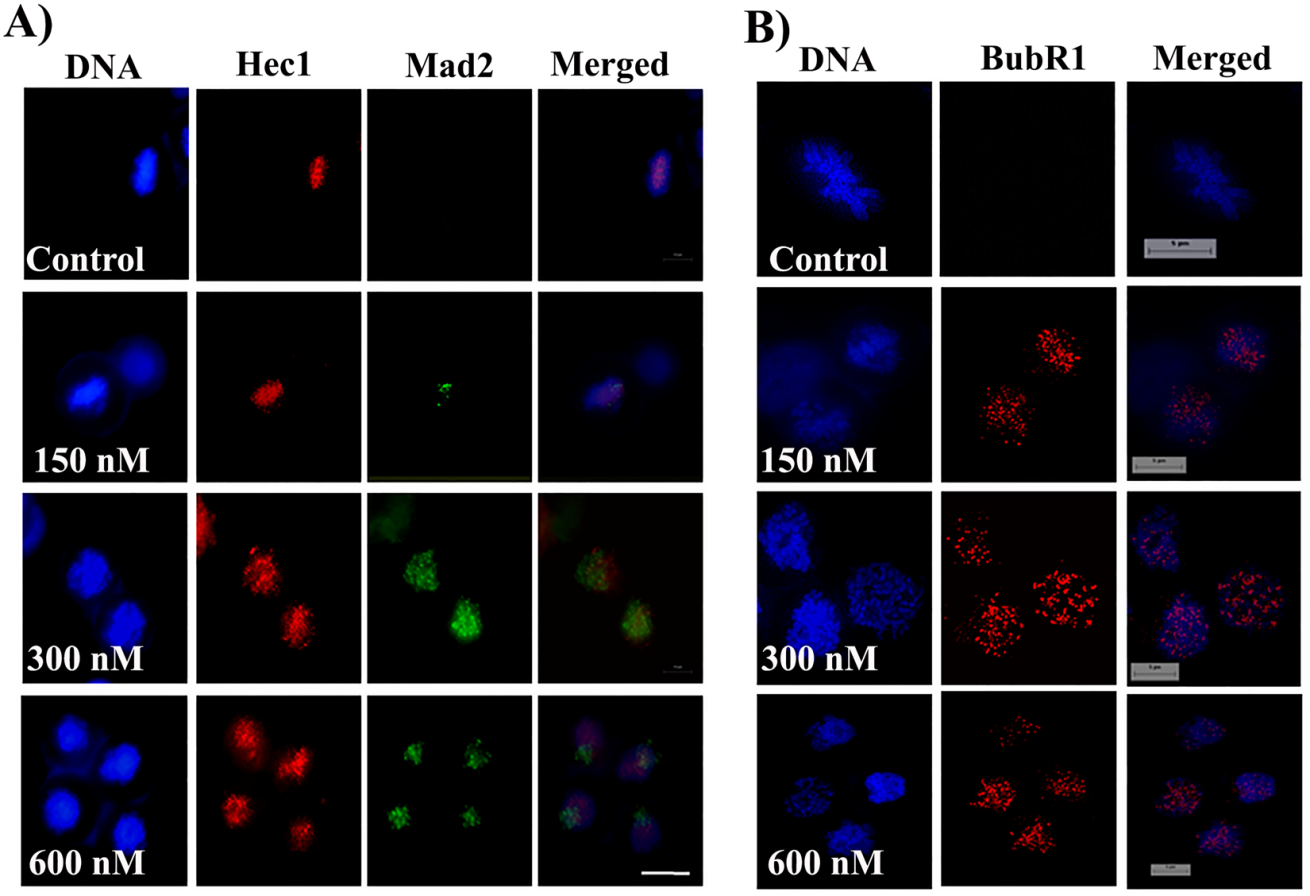
Indibulin activated the mitotic checkpoints in MCF-7 cells: (**A**,**B**) MCF-7 cells were incubated with a vehicle or different concentrations of indibulin for 24 h and processed for immunostaining with antibodies against Hec1 (red) and Mad2 (green) (**A**) or BubR1 (red) (**B**). Scale bar = 10 µm.

**Figure 6:**
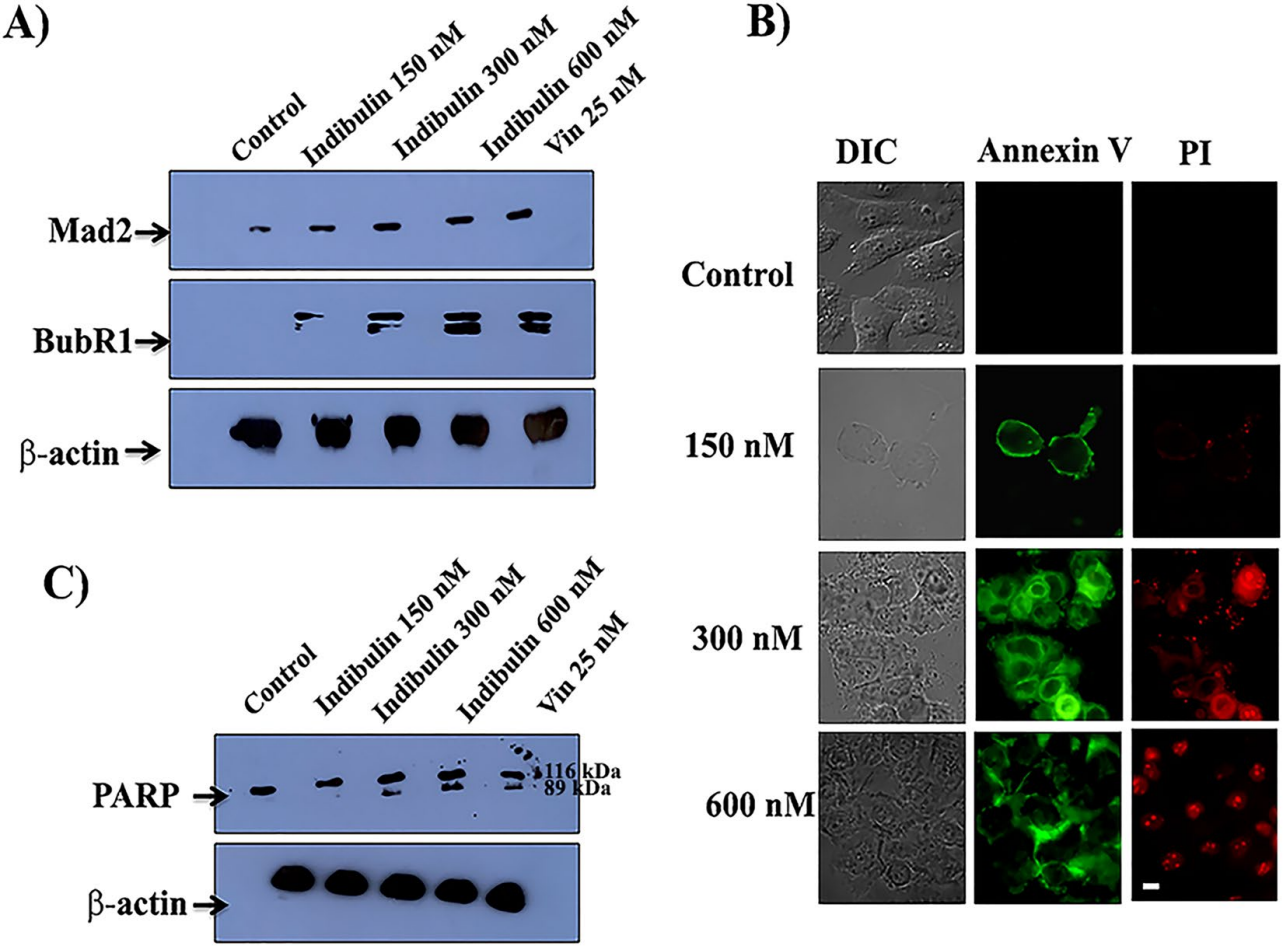
Indibulin increased the level of Mad2 and BubR1 and activated apoptosis in MCF-7 cells: (**A**) MCF-7 cells were incubated without and with different concentrations of indibulin and the cell extract was prepared. Vinblastine (25 nM) was used as a positive control. Western blot was performed with the extracts and immunoblotting was done with anti-Mad2 IgG, anti-BubR1 IgG, and anti-β actin IgG. The appropriate molecular weight bands of Mad2 (24 kDa), BubR1 (120 kDa) and β-actin (42 kDa) were cut from the same gel and immunoblotted with respective antibodies. The experiment was performed three times, shown is one of the representative blots. (**B**) MCF-7 cells were incubated with vehicle or different concentrations of indibulin for 48 h and then stained with annexin V (green) and propidium iodide (red) for detecting apoptosis. Scale bar = 10 µm. (**C**) Western blot of MCF-7 whole cell extracts prepared after incubating cells without and with different concentrations of indibulin and vinblastine (25 nM) for 48 h. The cell extract was separated on SDS-PAGE and appropriate molecular bands of PARP (116 kDa) and β-actin (42 kDa) were cut from the same gel. Immunoblotting was done with anti-PARP IgG and anti-β actin IgG. Shown is one of the representative blots from three experiments.

Mitotic checkpoint proteins Mad2 and BubR1 are shown to recognize proper microtubule-kinetochore attachment and kinetochoric tension and do not allow cells to divide until all kinetochores are attached properly to the microtubules^18^. As expected, in control cells, the chromosomes were aligned compactly on the metaphase plate and Mad2 and BubR1 were undetectable (Fig. 5A,B). On the other hand, in cells treated with indibulin, chromosomes were not aligned at the metaphase plate and Mad2 and BubR1 were found to localize at the kinetochoric region of a large number of mitotic cells. For example, 5, 38, 74 and 95% of the mitotic cells were found to be positive for BubR1 localization in the absence and presence of 150, 300, and 600 nM indibulin, respectively. The data indicated that indibulin perturbed the attachment of microtubules to kinetochores and did not allow proper kinetochoric tension to develop. The level of Mad2 and BubR1 in indubulin treated MCF-7 cell extracts was also quantified by Western blot (Fig. 6A). In the presence of 600 nM indibulin, the level of Mad2 was found to increase by 68 ± 10% as compared to the control cells. Vinblastine (25 nM) also increased the Mad2 level by 71 ± 6%. Both the phosphorylated and un-phosphorylated forms of BubR1 were found to increase in indibulin treated MCF-7 cells (Fig. 6A) indicating the activation of spindle assembly checkpoint proteins^19,20^.

We then determined if indibulin-induced mitotic block caused apoptotic cell death. Using annexin V/propidium iodide staining, indibulin-treated cells were found to be in different stages of apoptosis (Fig. 6B, Table 2). For example, 1.7 ± 1.2 and 23.3 ± 5.5% of the cells were in the late stages of apoptosis while 1.3 ± 0.6 and 29 ± 5% cells were found to be dead in the absence and presence of 600 nM indibulin (Table 2). Further, the cleavage of PARP protein was monitored to confirm apoptosis^21,22^. The treatment of MCF-7 cells with 300 and 600 nM of indibulin generated cleaved fragments of PARP protein indicating that indibulin-treatment induces apoptosis in MCF-7 cells (Fig. 6C). As reported earlier^23^, vinblastine treatment was also found to induce PARP cleavage in MCF-7 cells (Fig. 6C).

**Table 2:** Indibulin treatment induced apoptosis in MCF-7 cells.

	Annexin V positive (Early Apoptosis)	Annexin V + PI positive (Late Apoptosis)	PI positive (Dead)
Control	2.7 ± 1.5	1.7 ± 1.2	1.3 ± 0.6
Indibulin (150 nM)	27 ± 4	4.7 ± 0.6	5.2 ± 2.8
Indibulin (300 nM)	9.3 ± 4.7	16 ± 3	10 ± 2
Indibulin (600 nM)	10.3 ± 3.1	23.3 ± 5.5	29 ± 5

The % of cells in different stages of apoptosis is shown. Data represent mean ± SD.

### Indibulin treatment synergizes with vinblastine

**Figure 7:**
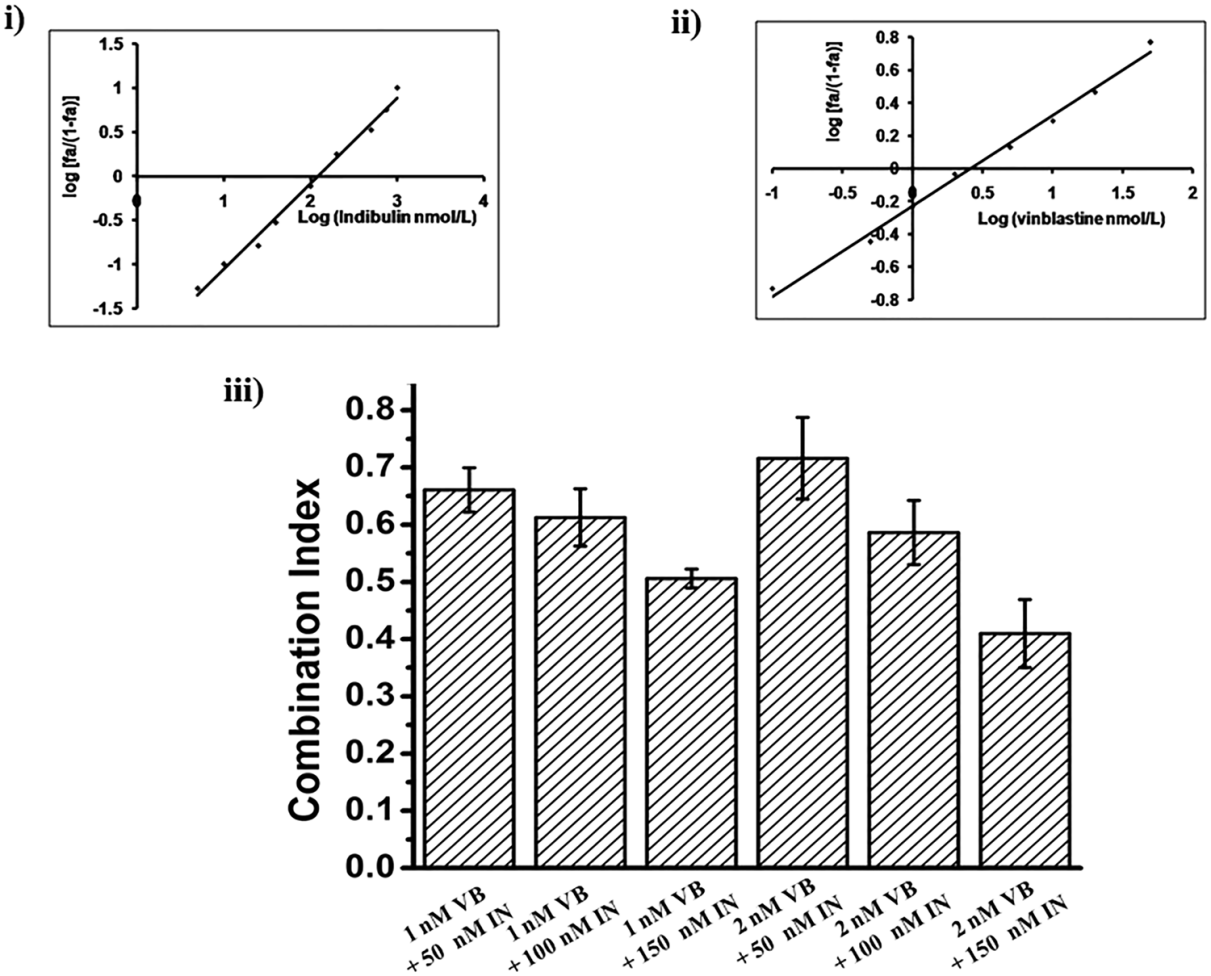
Indibulin and vinblastine exert synergistic anti-proliferative effects on MCF-7 cells. The median dose plot for the inhibition of cell proliferation in the presence of indibulin (i) and vinblastine (ii) is shown. Histogram (iii) shows the combination indices for the combination of indibulin (IN) with vinblastine (VB). Data are average of three independent experiments and represent mean ± SD.

Since indibulin does not bind to vinblastine binding site on tubulin^1^, we hypothesized that the combination of indibulin and vinblastine should exert either a synergistic or an additive effect on MCF-7 cells. The combination of indibulin (Fig. 7,i) and vinblastine (Fig. 7,ii) was found to produce a synergistic inhibitory effect on cell proliferation. The median inhibitory doses of indibulin and vinblastine were found to be 150 ± 13 and 2.2 ± 0.6 nM, respectively, (Fig. 7,i,ii). Indibulin (50 and 150 nM) in combination with 1 nM vinblastine inhibited the proliferation of MCF-7 cells by 53 and 71%, respectively, and produced a combination index (CI) of 0.67 ± 0.03 and 0.5 ± 0.02. A combination of 2 nM vinblastine and 100 nM indibulin inhibited the proliferation of cells by 67%, with a CI of 0.6 ± 0.06 while a combination of 2 nM vinblastine and 150 nM indibulin was also strongly synergistic with a CI of 0.41 ± 0.06.The combination indices for all the combinations of indibulin and vinblastine were found to be ≤1 (Fig. 7,iii) indicating that vinblastine and indibulin produced synergistic effects in inhibiting the proliferation of MCF-7 cells.

### Effects of indibulin on undifferentiated and differentiated neuroblastoma- SH-SY5Y cells

**Figure 8:**
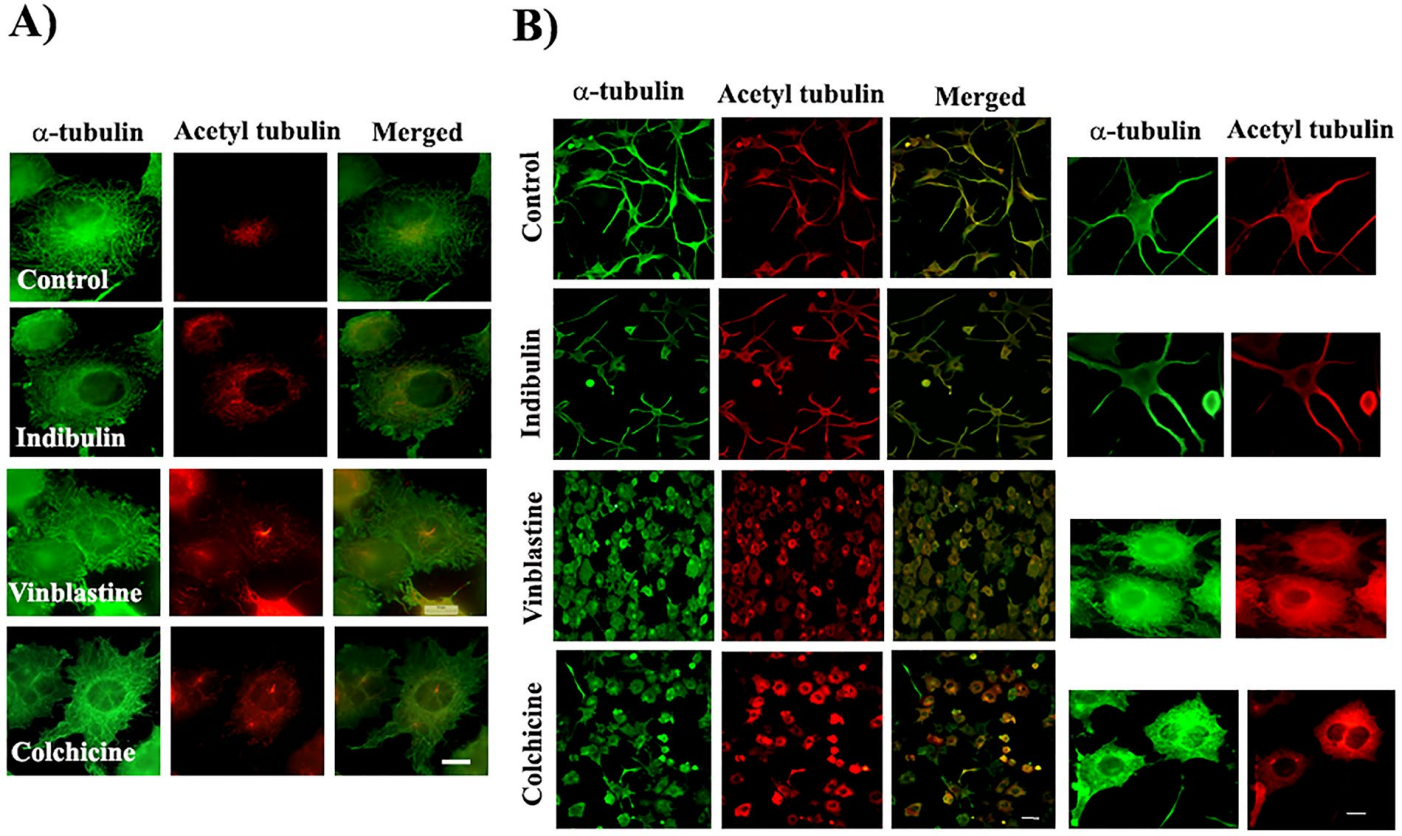
Effects of indibulin on undifferentiated and differentiated SH-SY5Y cells. (**A**) Undifferentiated SH-SY5Y cells were incubated in the absence and presence of indibulin (25 nM), vinblastine (10 nM) and colchicine (10 nM) for 24 h. Cells were fixed and processed for immunostaining with antibodies against α-tubulin (green) and acetylated tubulin (red). Scale bar = 10 μm. (**B**) Differentiated SH-SY5Y cells were treated and processed as in (**A**) and observed at two magnifications under a microscope. Scale bar = 100 and 10 μm in low (Left panel) and high (Right panel) magnification, respectively.

A previous study suggested that the acetylation of tubulin reduced the effects of indibulin on microtubules^24^. We hypothesized that the low neurotoxic effects of indibulin could be due to the high acetylation level of neuronal microtubules^24^. To verify this, the effect of indibulin on differentiated neuronal cells was determined. We differentiated the SH-SY5Y neuroblastoma cells as they are a suitable model for differentiated primary cells^25,26^. We first determined the effect of indibulin, vinblastine, and colchicine on the proliferation of undifferentiated SH-SY5Y cells. The undifferentiated SH-SY5Y cells behaved like other dividing cells and microtubules in these cells were found to be minimally acetylated. The compounds inhibited the proliferation of undifferentiated SH-SY5Y cells in a concentration-dependent manner and the half-maximal inhibitory concentrations of indibulin, vinblastine and colchicine were determined to be 25 ± 4, 9.3 ± 1.8 and 11 ± 3 nM, respectively. The effects of vinblastine, colchicine, and indibulin on microtubules in cells was then determined at their respective half-maximal inhibitory concentrations. The cells were found to be sensitive towards these agents (Fig. 8A). The microtubules in the cells treated with colchicine (10 nM), vinblastine (10 nM) and indibulin (25 nM) were found to be depolymerized. We then determined the effect of indibulin on the differentiated neuronal cells. Microtubules in the neurites, as well as cell body of differentiated neurons, were enriched in tubulin acetylation. In vehicle-treated neuronal cells, multiple large axon-like neurite extensions extending upto 100 μm were observed (Fig. 8B). When the differentiated cells were treated with 10 nM colchicine and 10 nM vinblastine, the microtubules were depolymerized and the differentiated phenotype of the cells was lost. Most of the differentiated cells retracted their neurite extensions. The total neurite length per neuron was found to reduce by 78 ± 8 and 86 ± 5% in 10 nM colchicine and vinblastine treated cells, respectively, as compared to that of the control cells. However, the cells treated with 25 nM indibulin retained their highly organized microtubule structures in neurites similar to the untreated control cells. The total neurite length per neuron was diminished by 12 ± 5% in indibulin-treated cells as compared to that of the control cells.The data indicated a significant reduction of the microtubule depolymerizing ability of indibulin in differentiated neuronal cells as compared that in the undifferentiated cells (Fig. 8B).

## Discussion

Indibulin has been shown to possess excellent *in vivo* antitumor activity in preclinical models and is undergoing further clinical evaluation in Phase II trials. In this study, we found that indibulin blocks mitosis by inhibiting microtubule dynamics. The combination of low doses of indibulin with vinblastine was found to be synergistic in inhibiting cell proliferation. It is quite possible that indibulin and vinblastine in concert with each other lead to much stronger effects on microtubule dynamics than their individual effects, resulting in strong synergism. The two drugs, thus, together may prove useful for combination therapy in the treatment of breast cancer.

### A possible mechanism for the antitumor effects of indibulin

Indibulin, at its effective cytotoxic concentrations, dampened dynamics of individual microtubules in live MCF-7 cells. Similar to vinblastine^27^, it affected the growth and shortening rates of microtubules. Indibulin significantly affected the length based catastrophe and rescue frequencies of microtubules. In addition, indibulin perturbed the localization of EB1, which is speculated to bind to microtubule plus ends by recognizing the GTP cap^16,17^. The data together indicated that indibulin altered the properties of microtubule ends. The dynamic instability of microtubules is important especially during metaphase for proper bi-oriented attachment and for the tension-associated oscillations of chromosomes^18^. A defect in these processes prevents the onset of anaphase by the mitotic checkpoint proteins that accumulate at kinetochores and act as a safety mechanism to ensure fidelity of chromosome segregation^18^. Although at its IC_50_ values, indibulin did not visibly depolymerize interphase microtubules, it exerted abnormalities like reduction in the spindle length and defects in the congression of chromosomes in the mitotic cells. As a result, even in the presence of low concentration (150 nM) of indibulin, the mitotic checkpoint proteins BubR1 and Mad2 were found to localize on the kinetochores in the mitotic cells. At 300 and 600 nM indibulin, where chromosome organization was visibly disrupted, large amounts of checkpoint proteins accumulated on chromosomes in MCF-7 cells. The suppression of microtubule dynamics by indibulin might prevent microtubules from capturing and aligning the chromosomes during the mitosis. The data together suggested that the antiproliferative activity of indibulin correlated well with its ability to produce multiple defects in spindle formation that inhibit the cell cycle progression at mitosis.

### Implications for neurotoxicity

A major disadvantage of microtubule inhibitors that severely impedes their continuous use in clinics and is often a dose-limiting complication is the development of neurotoxicity^28^. Paclitaxel and the first-generation *vinca* alkaloids and even the newer drugs like ixabepilone cause severe sensory and motor neuropathy, which might even result in termination of chemotherapy^29^. Indibulin was shown to lack neurotoxicity that is usually associated with other microtubule-targeted drugs^1,4,5^. An earlier study suggested that indibulin might discriminate between post-translationally modified and unmodified tubulin^24^. We found that the integrity of microtubules in differentiated SH-SY5Y neurites was comparatively less affected by indibulin while colchicine and vinblastine completely disrupted the microtubule structure in cells. Since indibulin could depolymerize microtubules in undifferentiated SH-SY5Y cells as effectively as colchicine and vinblastine, we ruled out the possibility that indibulin is not able to enter SH-SY5Y cells. Our data together with the previous report^24^ suggested that the unusually high level of acetylation in neuronal microtubules reduces the binding affinity of indibulin to tubulin rendering it less detrimental to neuronal cells. The results indicated that the combined use of indibulin with vinblastine can produce synergistic antiproliferative effects. Since indibulin has been shown to lack neurotoxicity^1,4,5^ while vinblastine is known to produce strong neurotoxicity^30–32^ a reduction in the dosage of vinblastine may help to reduce the neurotoxicity caused by the high dosages of vinblastine. The results also provide an appealing rationale for using indibulin as a lead molecule in structure-guided drug designing to developing new analogs possessing superior activity and lacking significant neurotoxicity.

## Materials and Methods

### Reagents

Indibulin was purchased from Tocris Biosciences. Sulforhodamine B (SRB), mouse monoclonal anti-α tubulin IgG, mouse monoclonal anti-β actin IgG, fluorescein isothiocyanate (FITC)-labeled anti-rabbit IgG, Hoechst 33258 and bovine serum albumin (BSA) were purchased from Sigma (St Louis, MO, USA). Mouse monoclonal anti-EB1 IgG, rabbit polyclonal anti-α tubulin IgG, fetal bovine serum (FBS), and retinoic acid were purchased from BD Transduction Laboratories (San Diego, CA, USA), Abcam (Cambridge, MA, USA), Biowest, Nuaille, France, and Calbiochem (NJ, USA), respectively. Alexa 568 conjugated anti-mouse IgG was purchased from Molecular Probes (Eugene, OR, USA). Anti-phosphohistone H3-S10 IgG and anti-rabbit PARP IgG was purchased from Santa Cruz Biotechnology (CA, USA). A chemiluminescent substrate (Super Signal West Pico) was obtained from Thermo Scientific (Rockford, USA).

### Cell culture and cell proliferation assay

Human breast cancer cells (MCF-7) were obtained from the cell repository of the National Centre for Cell Science, Pune, India. The repository confirmed the species by mt-rDNA sequencing. Human neuroblastoma (SH-SY5Y) cell line was a kind gift from Dr. BN Mallick, Jawaharlal Nehru University, India. The cell lines were found to be free of mycoplasma. The cells were cultured as described earlier^12,33^. For differentiation of SH-SY5Y cells, the cells were seeded at a density of 0.3 × 10^5^ cells/mL on glass coverslips in 24 well plates and were treated with all-trans retinoic acid for 48 h^25^. To determine the effect on the proliferation of cells, MCF-7 or SH-SY5Y cells were seeded at a density of 1 × 10^5^ cells/mL in 96 well plates. After the cells attached, MCF-7 or SH-SY5Y cells were incubated with either vehicle or different concentrations of indibulin, vinblastine or colchicine for 48 h or 24 h, respectively. The antiproliferative effect of indibulin, vinblastine or colchicine was determined by Sulforhodamine B assay^12,34^.

### Cell cycle analysis

MCF-7 cells were incubated without or with different concentrations of indibulin for 48 h. Samples for flow cytometry were prepared as described earlier^12^. In brief, the cells were fixed with 70% ethanol and then, incubated with propidium iodide (400 μg/mL) and RNaseA (1 μg/mL) for 2 h. The effects of indibulin on the cell cycle were monitored using a flow cytometer (FACS Aria special order system, Becton Dickinson, USA) and the data were analyzed by Modfit LT program (Verity Software, ME, USA).

### Immunofluorescence assay

MCF-7 cells were either incubated without or with different concentrations of indibulin and were then fixed and processed for immunostaining as described earlier^11,12,33,35^. The total number of cells were counted using Hoechst staining under the fluorescence microscope. Cells were examined using Nikon Eclipse TE2000-U fluorescence microscope and the images were captured and analyzed using Image-Pro Plus 4.5. The percentage of monopolar and multipolar cells in the mitotic population was determined by scoring 200 mitotic cells in each experimental condition. The interpolar distance was determined by measuring the distance between the spindle poles as indicated by γ-tubulin^14^. The mean interpolar distance was determined by scoring 20 bipolar spindles in each condition. In a separate assay, cells were treated with vehicle or different concentrations of indibulin for 48 h and then fixed and stained with antibodies against α-tubulin, phosphohistone H3 (S10), Mad2 or BubR1. DNA was stained with Hoechst. The experiment was performed three times. The percentage of positive BubR1 cells was estimated by scoring 200 mitotic cells in each experimental condition. Images were collected using Nikon Eclipse TE2000-U fluorescence microscope. For detecting apoptosis, annexin V/propidium iodide assay was used^21,36,37^. MCF-7 cells were treated with vehicle or different concentrations (150, 300 and 600 nM) of indibulin for 48 h and subsequently, the cells were stained with annexin V and propidium iodide. The cells are scored as early apoptotic, late apoptotic and dead based on only annexin V, both annexin V and propidium iodide and only propidium iodide staining, respectively^21,36,37^. In each experimental condition, 200 cells were scored to estimate the number of apoptotic cells. The neurite length in differentiated SH-SY5Y cells was measured using ImageJ as described earlier^38^.

### Western blotting

MCF-7 cells were seeded at a density of 1 × 10^6^ cells/mL in tissue culture flasks. When 70% confluency was reached, the cells were incubated without or with different concentrations of indibulin or 25 nM vinblastine for 48 h. The whole cell lysate or tubulin polymeric fraction was prepared. The soluble and polymeric fraction of tubulin was separated^39^. Briefly, PEM buffer (50 mM PIPES, 3 mM MgCl_2_ and 1 mM EGTA) containing 0.5% Triton-X-100 and 25% glycerol was added to the cell pellet for 2 min to collect soluble tubulin at 37 °C. Insoluble tubulin (polymerized MT) was collected by treating the pellet with cell lysis buffer (25 mM Tris–HCl (pH 7.6), 200 mM NaCl, 1% NP-40, 1% sodium deoxycholate and 0.1% SDS) containing protease inhibitors for 1 h at 4 °C. Equal amounts of protein were loaded on 12% SDS-PAGE and then were subjected to Western blotting on a PVDF membrane. The membrane was incubated with either anti-α tubulin IgG, anti-Mad2 IgG, anti-β actin IgG or anti-BubR1 IgG. The band intensity was estimated by ImageJ. The experiment was performed thrice in each case.

### Transfection and Time-lapse imaging

EGFP-tubulin plasmid was transfected into MCF-7 cells using Lipofectamine 2000 following manufacturer’s protocol. Transfected cells were maintained in the presence of G418 and seeded on glass coverslips before the experiment. Cells were treated with indibulin for 3 h. Microtubule dynamics in live MCF-7 cells was monitored using a laser scanning confocal microscope with 60x oil immersion objectives (FluoView 500 Olympus, Tokyo, Japan). The plus ends of microtubules were tracked using ImageJ^11,12^. Microtubule dynamic instability parameters were calculated as described earlier^11,12,15^.

### Determination of Combination Index

MCF-7 cells were treated with 1 and 2 nM vinblastine, or 50, 100 and 150 nM indibulin individually or with the combination of indibulin and vinblastine for 48 h. The effect on the proliferation of MCF-7 cells was measured by Sulforhodamine B assay and the combination index was calculated by the Chou and Talalay method^40^ as described earlier^41^.

### Statistical analysis

The experiments were carried out at least three times. Data are presented as mean ± SD. Data were analyzed using one-way ANOVA using the Origin 7.5 software.

### Data availability

All data presented in the study are included in the manuscript as figures and tables.
